# A Collaborative Communities-of-Practice Methodology for Environmental Design Research: A Case Study Application in Palliative Care

**DOI:** 10.1177/19375867251365876

**Published:** 2025-08-28

**Authors:** Emil E. Jonescu, Benjamin Farrell, Chamil Erik Ramanayaka, Lori Delaney, Edward Litton, Talia J. Uylaki, Gareth Watts, Bella Brigham, Frances Bellemore, Davinia Seah

**Affiliations:** 1 95980 Edith Cowan University, Australia; 2 Hames Sharley (Perth), Australia; 3 Gabriels Hearne Farrell Pty Ltd, Australia; 4 6939 Central Queensland University, Australia; 5 7932 University of Southern Queensland, Australia; 6 85075 Fiona Stanley Hospital, Australia; 7 UWA, Australia; 8 St John of God Hospital Subiaco, Australia; 9 Hames Sharley (Adelaide), Australia; 10 7800 Royal Prince Alfred Hospital, Australia; 11 University of New South Wales, Australia; 12 2787 St Vincent's Hospital Sydney, Australia; 13 523002 University of Notre Dame Australia, Australia

**Keywords:** interdisciplinary research, communities of practice, healthcare design methodology, palliative care environments, mixed-methods research

## Abstract

Environmental design in palliative care settings receives increasing attention, yet methodologies for studying such environments often lack interdisciplinary integration. Traditional research designs may overlook the lived expertise of clinicians and designers.

**Objectives:**

This article describes a collaborative, cross-disciplinary communities-of-practice model developed as a methodological framework to enable effective environmental evaluation and design processes in healthcare settings. Its application is illustrated through a case study in a palliative care unit.

**Methods:**

A co-designed, multi-methods approach was developed by a team of clinicians, facility managers, and academic researchers. The methodology included a scoping review, site-specific environmental data logging (sound, temperature, lighting), and co-created survey tools for staff and patients or proxies. Data were collected over two phases, following ethically approved protocols to protect privacy and support data validity.

**Results:**

The communities-of-practice model successfully integrated cross-sector expertise, improved the contextual relevance of study instruments, and enabled real-time, context-sensitive data collection in a high-acuity clinical setting. The method supported context-aware adaptations that would not have emerged from conventional top-down research approaches, effectively bridging academic inquiry and practical clinical application.

**Conclusion:**

The communities-of-practice model offers a replicable, interdisciplinary method for researching complex healthcare environments. Its case study in a palliative care unit demonstrates its capacity to generate actionable insights aligned with patient-centred outcomes. As health architecture increasingly intersects with evidence-based care delivery, such methodological approaches are vital for aligning design decisions with clinical and human-centred goals.

## Introduction

Palliative care units (PCUs) represent places where clinical expertise, emotional care, and architectural sensitivity intersect. In these highly specialised settings, environmental quality acts both as a backdrop to care and as an active contributor to wellbeing and performance for patients, families and healthcare professionals. The palliative care environment must accommodate the unique needs of individuals approaching the end of life, who are often characterised by physical frailty, cognitive impairment and emotional vulnerability. As such, it presents a significant design challenge requiring insights that extend beyond traditional healthcare engineering or narrowly clinical considerations (Jonescu, Farrel et al., 2024; Jonescu et al., 2025; Tishelman et al., 2016). Australian contemporary healthcare standards underscore the role of the built environment in supporting patient outcomes, advocating for non-pharmacological interventions and diurnal lighting patterns that replicate natural daylight to promote sleep and reduce complications such as delirium (Australian Commission on Safety and Quality in Health Care, 2021). This evidence highlights the critical nature of environmental conditions as essential components of care delivery.

Despite these imperatives, most empirical studies in healthcare design continue to adopt narrowly framed methodologies that exclude the lived expertise of frontline staff or the practice-informed knowledge of environmental design professionals. Research methods that prioritise disciplinary control over collaborative exploration risk overlooking crucial contextual variables and fail to deliver findings that translate effectively to real-world practice (Greenhalgh et al., 2016). Communities of Practice (CoP) frameworks offer an alternative. Developed initially as a social learning theory (Wenger, 1998), CoP models emphasise mutual engagement, joint enterprise, and a shared repertoire of knowledge across stakeholder groups. When adapted for healthcare contexts, CoPs facilitate the development of shared understanding and co-constructed solutions among clinicians, architects, designers, and researchers (Li et al., 2009).

This article describes a collaborative communities-of-practice (CoP) methodology designed to support interdisciplinary research and design in complex healthcare environments. The method is presented as a replicable framework, with its application demonstrated through a case study of a PCU. The CoP-based approach integrated clinicians, healthcare designers, built environment researchers, and technical experts to co-develop a robust mixed-methods research design. This included environmental data collection on sound levels, light, and temperature, in combination with staff and patient-proxy surveys, to evaluate the cumulative and concurrent effects of environmental conditions on wellbeing and performance. The emphasis throughout was on aligning empirical data with clinical priorities and design feasibility.

This article forms one of several outputs from a broader interdisciplinary study, with related manuscripts currently under review or in preparation and addressing other distinct research questions for specific professional audiences. By foregrounding the collaborative CoP methodology, this article contributes to the expanding field of evidence-based healthcare design. It demonstrates how rigorous interdisciplinary methods can be developed to balance academic credibility with practical, context-sensitive implementation, thereby strengthening the translation of research findings into tangible improvements in PCU environments and similar healthcare settings.

## Background

Environmental quality in healthcare has long been recognised as a determinant of health outcomes, particularly in high-dependency settings. The theory of supportive design posits that environments capable of reducing stress can enhance patient recovery, support staff efficiency, and foster a sense of control and comfort (Ulrich, 1991). Subsequent work has reinforced this principle, demonstrating links between spatial design features and reduced agitation (Marquardt et al., 2014), lower noise levels and improved communication (Busch-Vishniac & Ryherd, 2023) and better lighting and patient sleep–wake cycles (Blume et al., 2019). However, research into the effects of environmental conditions within PCUs remains limited. While intensive care units and emergency departments have been extensively studied (Delaney et al., 2017; Litton et al., 2017), PCUs differ in their patient profile, therapeutic goals, and psychosocial atmosphere that is conducive to patients nearing the end of life. Disruptions to patient care can significantly impact physical, emotional and sensory aspects of the patient and staff experience. These include staff and patient vocalisations, ambient sounds from equipment and alarms, fluctuating lighting and frequent clinical interventions thus methodological approaches must be able to account for all aspects of the care environment (Agom et al., 2022; Fridh et al., 2009; McLaughlan & George, 2022; McLaughlan & Richards, 2023; Miller et al., 2023).

Environmental stressors such as poor acoustic control, unstable thermal conditions and inadequate lighting result in conditions that are counterproductive to a not conducive to a sleep supportive environment, general sense of satisfaction and wellbeing for both patients and clinical staff, aspects that are critical to the integration of holistic care (Czeisler & Buxton, 2005; Lee et al., 2021; Miller et al., 2023; Okamoto-Mizuno & Mizuno, 2012; Ormandy & Ezratty, 2012, 2015; Ye et al., 2012). A sleep-supportive environment stems from the convergence of a multiple environmental parameters, such as light exposure and ambient temperature that influence the body's circadian rhythm, and sound levels which affect sleep continuity, arousal thresholds and overall sleep quality. In palliative care settings, where symptom management and quality of life are central, growing evidence shows that addressing sleep disturbances through environmental design is essential to improving patient comfort, reducing distress and enhancing care outcomes (Jeon et al., 2024).

The literature reviewed in this study identifies and informed the environmental thresholds considered optimal for maintaining a high-quality physical environment within the PCU. For circadian rhythm regulation and daytime alertness, light exposure of 1,000–2,000 lux for at least 30–60 minutes during the daytime, alongside a maximum melanopic equivalent daylight illuminance (EDI) of 10 lux in the evening, has been recommended (Thompson et al., 2012). Thermal comfort literature suggests an ambient room temperature range of 18–22 °C, with a diurnal variation greater than 2 °C and a gradual reduction in temperature overnight to support thermoregulation during sleep (Okamoto-Mizuno & Mizuno, 2012). In terms of acoustics, Australian Standards (AS 2107:2016) recommend equivalent continuous sound levels (Leq) of 35–40 dB(A) for patient bedspaces. The World Health Organization guidelines further suggest Leq levels not exceeding 30 dB(A) at all times and nighttime maximum sound pressure levels (Lmax[F]) below 40 dB(A) (Berglund et al., 2010; World Health Organization, 1999). However, more recent findings indicate that internal maximum sound levels below 50–55 dB(A) are unlikely to result in sleep disturbance or arousal (Jonescu, Farrel et al., 2024). These stressors have been linked to staff burnout, communication breakdowns and decreased care quality (Higashibata et al., 2023; Manias et al., 2017, 2019; Sundberg et al., 2017). Despite this, few studies integrate these factors into a single methodological framework, and even fewer engage practitioners across disciplines in the co-design of research instruments and interpretative strategies.

With the aim of developing a methodology capable of quantifying commonly under-addressed environmental determinants of sleep quality, and examining their individual and combined alignment with evidence-based thresholds for sleep-promoting conditions, this study sought to address a critical gap in healthcare design research. A CoP framework was adopted to guide the development and implementation of an environmental performance evaluation, using a PCU as a case study to illustrate its application. This approach facilitated transdisciplinary collaboration and mutual learning across clinical, academic, and design domains. By doing so, the study reflects an epistemological shift toward more situated, practice-informed, and ethically responsive modes of inquiry in healthcare research.

The CoP methodology supported co-creation of methods, iterative refinement of research tools, and grounded interpretation of data in ways that traditional disciplinary silos often prevent. This project builds on calls for more integrative approaches in health services research (Greenhalgh et al., 2016; Kitson et al., 2017), aiming to reshape how evidence is gathered, validated and translated for sensitive clinical environments.

## Project Approach and Methodology

This study adopted a novel transdisciplinary methodological framework grounded in the CoP model (Wenger, 1998), bringing together clinicians, industry practitioners and academic researchers to co-produce knowledge relevant to healthcare design research. The integration of diverse professional perspectives was central to both the development and implementation of the framework, supporting ecological validity, translational relevance and operational feasibility. This approach also responded to broader calls for embedded research models in healthcare environments that aim to bridge the gap between design theory and clinical realities (Jonescu, Farrel et al., 2024).

The project was structured in two distinct phases. Phase A involved a scoping review of the existing literature to inform the study's aims and objectives and to identify limitations and exclusions in prior work. Phase A also included the co-design of two structured surveys: one targeting clinical and ancillary staff working within a metropolitan Australian PCU, to be administered during Phase A, and another directed towards patients (by proxy) to be administered in Phase B.

The survey instruments were collaboratively developed by environmental design researchers, sleep specialists, and healthcare practitioners, and underwent multiple rounds of review to maximise clarity, face validity, and relevance to practice. Both surveys employed a 7-point Likert scale (1 = strongly disagree; 7 = strongly agree), supplemented by open-ended questions to capture qualitative insights. The staff survey explored perceptions of environmental conditions, including sound levels, temperature, and lighting, and their effects on patient care and staff performance. Inclusion criteria were restricted to staff members employed in the ward for at least three months to ensure familiarity with the environment.

The patient and by-proxy survey assessed perceptions of environmental conditions, their impacts on patient experiences, and offered a more comprehensive understanding of how these factors influence the inpatient journey. The study sought to identify potential cross-correlations between survey results and data derived from environmental monitoring. Inclusion criteria required participants who had spent at least two nights on the ward and had sufficient time to complete the survey. Exclusion criteria included lack of English proficiency, non-primary caregivers, proxies involved in the patient's care for less than one month, those with cognitive or mental health conditions affecting response accuracy, individuals with potential conflicts of interest, and proxies from whom informed consent could not be obtained. Proxies providing information inconsistent with the medical record were also excluded. For example, proxies who reported the patient was physically active despite records documenting bedbound status during the same period were excluded to protect data accuracy.

**Figure 1.**
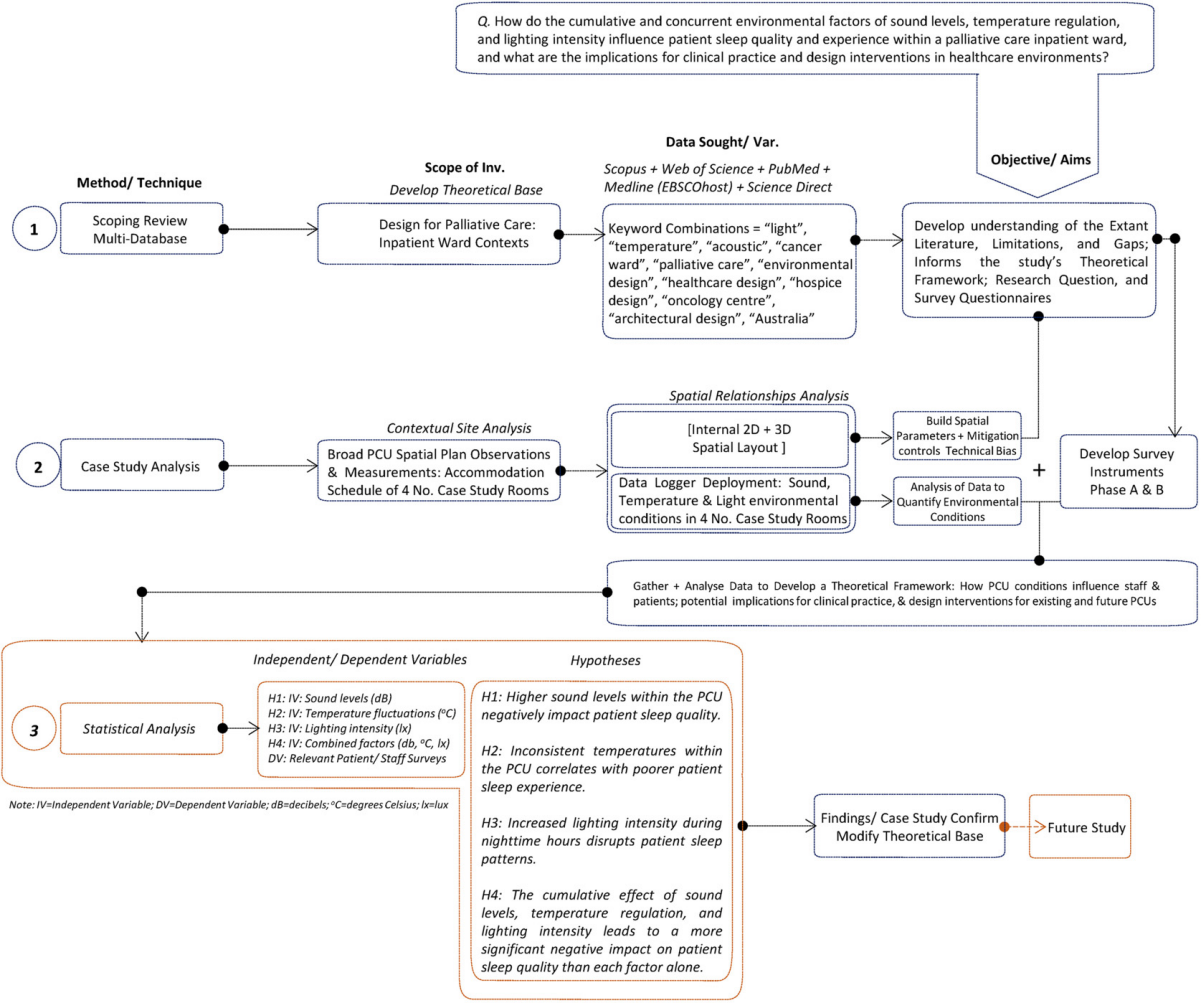
Diagram of research methods and their relationships.

To complement and validate staff observations, Phase A also involved the placement of calibrated environmental monitoring instruments to collect real-time data on acoustic levels, lighting intensity, and temperature across two occupancy cycles. The use of Type 1 sound level meters and lux/thermal dataloggers ensured compliance with ISO and ANSI standards (International Electrotechnical Commission, 2013; International Organization for Standardization (ISO), 2008). Devices were positioned in consultation with clinical staff to avoid hindering clinical processes in both single- and multi-bed rooms and programmed to record at one-minute intervals. Data were exported and analysed to establish environmental baselines and variation patterns in relation to ward design features. Figure 1 illustrates the relationship between research methods.

Ethical approval was obtained from the Human Research Ethics Committee (HREC Approval #2024/ETH01551). All participants were recruited voluntarily, provided informed consent, and were assured of the confidentiality and anonymity of their responses. The study adhered to principles of participatory ethics (Green & Thorogood, 2018), particularly in its engagement with frontline clinicians and patients (by proxy). The CoP framework used here aligns with calls for collaborative, embedded research methodologies in health design (Jonescu, Farrel et al., 2024; Jonescu et al., 2025; Neves et al., 2021; Rowe et al., 2020).

Rather than approaching built environment studies as externally imposed evaluations, this methodology positions stakeholders as co-inquirers and knowledge-holders, supporting iterative reflection and design validation during use. By integrating sensor-based environmental data with staff-driven insights and collaborative interpretation, this framework promotes inclusive, evidence-informed design that better aligns with the functional and emotional demands of palliative care settings. This methodological approach also strengthens the internal validity of findings by triangulating quantitative, qualitative, and practice-based data sources.

The synthesis of multidisciplinary perspectives ensures that proposed design implications are not only technically and clinically feasible, but also organisationally and culturally sustainable. Through this CoP-based framework, the study contributes to a growing methodological principle of embedded, co-produced health design research and offers a scalable template for future PCU-related inquiries and broader policy interventions.

## Findings and Discussion

### Introduction to Environmental Complexity and Interdisciplinary Response

Physical site observations preceding the formal data collection phase revealed a complex acoustic environment within the functioning PCU. This soundscape was shaped by overlapping sources, including clinical equipment (oxygen tanks, monitors, wheeled trolleys), human activity (staff movements, patient vocalisations, family conversations), and infrastructural systems (ventilation, alarms, telecommunications). These interacting elements produced a variable and multi-layered acoustic signature, modulated by the spatial configuration and material properties of the PCU's design. The resulting acoustic environment influenced both routine operational functions and the therapeutic processes of the ward. These observations are consistent with prior studies describing the heightened sensory and social complexity of healthcare soundscapes (Busch-Vishniac et al., 2005; Gaminiesfahani et al., 2020).

From a methodological standpoint, this preliminary environmental analysis reinforced the importance of a transdisciplinary research approach that unites clinical practitioners, architects, acoustic engineers, and environmental psychologists. The integration of expertise across these domains enabled the research team to collaboratively frame the design of the study and identify variables of clinical and operational relevance, particularly light, temperature, and sound, which are often underrepresented in conventional clinical trials (Rowe et al., 2020; Ulrich et al., 2008).

### Formation and Function of the Community of Practice

The research methodology was shaped by the recognition that environmental challenges in healthcare represent a multidisciplinary problem, one that resists resolution through any single-discipline lens. To address this, the study adopted a CoP framework, highlighting the value of synergistic knowledge generation through collaborative engagement. Stakeholders including ward-based clinicians, academic researchers, technical consultants and design professionals collectively contributed to framing research questions, refining data collection protocols and interpreting findings.

This transdisciplinary approach proved essential for deploying instrumentation effectively and for integrating diverse data streams. The CoP model supported a nuanced interpretation of findings, allowing the team to distinguish clinically and contextually relevant patterns from background variability, and to formulate actionable insights that would likely have been unattainable through a single-disciplinary methodology. According to Wenger (1998) a CoP framework focuses on learning through shared domains, community, and practice, underpinned all study phases.

Application of this method demonstrated its capacity to reveal latent environmental challenges as well as opportunities for operational improvement. For instance, the study identified inconsistencies in night-time lighting that may interfere with circadian rhythm regulation, and spatial incongruities such as the proximity of nurse stations to patient rooms that can contribute to avoidable disturbances. Importantly, the research highlighted how environmental indicators, including lighting, temperature and sound levels, influence staff and patient perceptions, communication effectiveness, subjective comfort and overall wellbeing. These insights point to a more holistic model of care in which the built environment acts as an active contributor to caregiving. However, they also reveal the challenge of balancing staff proximity, critical for timely intervention and fall prevention, with the preservation of sleep quality, which can be undermined by the light and sound associated with increased clinical surveillance. These results underscore the necessity of integrated environmental design strategies to support both clinical operations and the therapeutic milieu within palliative settings.

Examples of applied CoP value included:

- Collaborative co-design of staff and patient (by proxy) survey instruments that ensured relevance to PCU frontline experience and organisational priorities.
- Site-specific acoustic logger placement determined in consultation with the Nurse Unit Manager to balance research needs with clinical workflow integrity.
- Review and interpretation of environmental data such as sound level spikes with clinical processes, enabled iterative improvements in data capture strategy and clinician feedback.
- A richer interpretation of survey responses through triangulation: clinicians provided context around patient feedback within workflows, design professionals linked comfort-related responses to spatial and environmental features, and researchers ensured analytical rigour.

These examples illustrate how interdisciplinary collaboration enriched both methodological rigour and operational sensitivity, a vital principle when researching sensitive environments such as PCUs (Neves et al., 2021).

### Early Thematic and Quantitative Integration

Although the findings here are expanded in subsequent papers, preliminary convergence between environmental monitoring and stakeholder insights revealed critical patterns. For instance, staff surveys indicated that environmental sound and lighting influenced their ability to deliver holistic care and communicate effectively. These insights aligned with sensor data that showed consistent night-time lux exceedance and LAmax levels surpassing 55 dB(A) over 60% of the time in all monitored rooms.

Such findings, derived through co-produced instruments and interdisciplinary reflection, validate the methodological strengths of a CoP framework. They also demonstrate the epistemological advantage of integrating multiple perspectives; clinical, spatial and technical to interpret how environmental qualities shape care delivery, perception and experiences.

## Discussion and Recommendations

This study introduced and applied a novel methodological framework grounded in a CoP model to guide post-occupancy evaluation (POE) within a sensitive healthcare environment. By assembling a cross-disciplinary cohort of clinicians, healthcare managers, architects, and academic researchers, the study demonstrated how collaborative knowledge production can generate more contextually meaningful findings. The CoP model, initially described as a structure for situated learning (Wenger et al., 2002), was here expanded to underpin both the design and implementation of a robust research method. This aligns with Beckett et al. (2018), who argue that co-produced research can navigate complexity and achieve transformative impacts through methodological integration. Its application enabled deeper engagement with clinical realities while supporting empirical evaluation of environmental parameters, namely sound levels, light and temperature, that influence patient wellbeing, staff performance and care delivery.

Unlike conventional paradigms that separate research design from clinical application, this methodology enabled a reciprocal flow of insight between theory and practice. Iterative consultation with stakeholders informed survey instrument design, while staff feedback on sensor placement and study timelines reduced disruption and improved data validity. This co-constructed approach ensured that the resulting environmental data and experience-based insights were technically robust and operationally grounded. Such a model is particularly critical in palliative contexts, where patients and staff are highly sensitive to environmental stressors and where rigid, data-centric methods without stakeholder involvement risk invalid or ethically problematic results (Bullen et al., 2014; Seymour et al., 2005).

The strength of the approach lies in its integration of objective sensor data with subjective experiential feedback from clinician and patient/proxy surveys. This multiphase strategy generated a depth of insights that are being developed into a series of follow-up empirical papers, each addressing distinct thematic and methodological angles. The combination of mixed methods, cross-disciplinary expertise and iterative stakeholder engagement aligns with best practice in healthcare design evaluation, particularly in end-of-life care, where ethical sensitivity, operational realism and translational relevance are paramount (Joseph & Ulrich, 2007; Mills et al., 2010).

This methodological model offers a replicable framework for future research not only in palliative care but across other high-acuity domains, including intensive care (Jonescu, Farrel et al., 2024; Jonescu et al., 2025), aged care, paediatrics and mental health (Jonescu, Chirichilli et al., 2024). Its success demonstrates the feasibility and value of embedding POE research into live clinical environments without compromising care delivery and highlights the benefits of fostering sustained partnerships between healthcare institutions and academic researchers.

To ensure broader adoption of this approach, several practical and policy-level recommendations are offered. First, the CoP framework should be institutionalised as part of healthcare infrastructure projects, embedding design researchers and clinical practitioners in shared evaluation teams responsible for guiding facility development and continuous improvement. Second, ethics committees and research funders should encourage or require interdisciplinary composition and stakeholder involvement in environmental health studies. Third, survey instruments and environmental assessment protocols should be co-designed with stakeholders to ensure broad relevance, conceptual clarity and ethical appropriateness.

Finally, POE should be mandated for all new healthcare developments and major refurbishments, with particular emphasis on departments such as palliative care, where environmental quality directly influences dignity, comfort and clinical outcomes.

In summary, this study provides a methodological roadmap for collaborative, transdisciplinary, and ethically grounded environmental research in complex healthcare contexts. The approach affirms best practice in evidence generation by integrating methodological diversity, stakeholder co-design and triangulation to ensure outcomes are both scientifically valid and operationally actionable.

## Limitations

While this study offers a comprehensive methodological framework grounded in cross-disciplinary collaboration, several limitations should be acknowledged. First, the research was conducted within a single acute PCU in a metropolitan hospital, which may limit the generalisability of the findings to other institutional, regional or cultural contexts. The environmental characteristics, organisational structures and staffing patterns in this setting may not fully reflect those of other healthcare environments.

The core contribution of this work, however, lies not in its representativeness but in the design of its pragmatic methodological approach. The CoP framework and mixed-methods structure are inherently adaptable, making them well suited to broader application across healthcare settings, including aged care, intensive care and mental health. As such, this approach may be viewed as both a model for high-context qualitative inquiry and as a quality assurance methodology capable of informing localised POE and practice refinement.

Finally, this work has potential relevance for healthcare planning and policy, particularly in informing future revisions of the Australasian Health Facility Guidelines. It highlights how co-produced environmental research can offer decision-makers robust, grounded evidence to support design, retrofit and operational improvements in settings where dignity, comfort and clinical excellence must coexist.

## Conclusion

This study presents a robust, co-produced methodological framework grounded in a CoP model for evaluating environmental and clinical dimensions in healthcare design. By embedding cross-disciplinary collaboration among clinicians, industry practitioners and academic researchers from project inception through to data analysis, the methodology enabled the production of highly contextualised, operationally relevant and ethically grounded knowledge. Unlike siloed research designs, the CoP approach supported iterative refinement of instruments, integration of mixed-methods data and the translation of environmental conditions into actionable clinical and design insights.

The strength of this methodology lies in its capacity to bridge the empirical rigour of environmental measurement with the human sensitivity required in complex care settings. Its application within a PCU illustrates how the framework can generate nuanced understandings of how light, sound levels and temperature affect both care delivery and patient wellbeing, while simultaneously advancing a replicable model of interdisciplinary research. The study reinforces the idea that method is not neutral; how research is conducted shapes what is learned, who is empowered and how findings are implemented. In the context of healthcare systems increasingly challenged by demographic shifts and resource constraints, this methodological approach offers a scalable and responsive path forward for future research and facility development.

Ultimately, the study affirms that designing for dignity, clinical excellence, and environmental responsiveness begins with the way we construct our investigations. This work provides a methodological blueprint for future research that is scientifically rigorous and deeply attuned to the realities of care in complex healthcare environments.

## Implications for Practice

This research has implications for practice in several ways:

- Integrating communities-of-practice frameworks into design research enables more relevant and ethically sound facility evaluation in sensitive care settings.
- Collaborative co-design of environmental assessment tools with clinicians improves instrument validity and supports staff engagement in evidence-based design processes.
- Combining sensor-based data with staff and patient perspectives provides richer, context-sensitive insights to inform actionable design improvements.
- Applying this methodology during post-occupancy evaluation can identify latent environmental stressors that affect patient sleep, comfort, and staff workflow.
- Designers and facility managers can use this framework to embed continuous improvement processes directly within clinical environments, supporting dignity and quality of care.

## Highlights

- Novel CoP-based methodology for healthcare environmental research.
- Demonstrated in a live palliative care unit (PCU).
- Integrates sensor data with staff and patient-proxy perspectives.
- Supports actionable, context-sensitive design improvements.
- Provides a replicable blueprint for future post-occupancy evaluations.
